# Rare renal tumor: a case report of juxtaglomerular cell tumor and literature review

**DOI:** 10.3389/fonc.2025.1648756

**Published:** 2025-09-30

**Authors:** Cheng Zhu, Zhong Tian, Yingfang Zhang, Tingting Yang, Zhongcong He, Shicheng Chen, Bo Yu, Neng Zhang, Ni Fu

**Affiliations:** ^1^ Department of Urology, The Second Affiliated Hospital of Zunyi Medical University, Zunyi, China; ^2^ Department of Nursing, The Affiliated Hospital of Zunyi Medical University, Zunyi, China; ^3^ Department of Pathology, The Affiliated Hospital of Zunyi Medical University, Zunyi, China; ^4^ Department of Urology, The Affiliated Hospital of Zunyi Medical University, Zunyi, China

**Keywords:** juxtaglomerular cell tumor, hypertension, rare disease, differential diagnosis, case report, review

## Abstract

Juxtaglomerular Cell Tumor (JGCT) is an extremely rare neoplasm of the kidney that poses a significant clinical challenge in terms of accurate diagnosis. The key to successful treatment lies in the accurate identification of renal lesion. Excessive secretion of renin by JGCT causes activation of renin-angiotensin-aldosterone system (RAAS) secondary to uncontrollable hypertension, hypokalemia and consequently a range of clinical manifestations. While most JGCTs are benign, there have been reports of malignant cases, thus requiring close follow-up. In this case report, the subject is a middle-aged female patient who has suffered from recurrent poorly controlled blood pressure for a number of years. Following a medical examination, the patient was found to have the right renal mass, which was pathologically confirmed to be JGCT after laparoscopic partial right nephrectomy. Thereafter, the patient’s blood pressure recovered steadily during the subsequent follow-up period. Furthermore, a comprehensive summary of the diagnosis, differential diagnosis, treatment and review of case reports of JGCT from the last decade is provided, encompassing malignant biological behaviors.

## Introduction

Juxtaglomerular Cell Tumor (JGCT) is an extremely rare renal tumor first described by Robertson et al. in 1967 (1) and formally named by Kihara et al. in 1968 (2), which has led to the disease being referred to as Robertson-Kihara syndrome. The tumor originates from the juxtaglomerular apparatus and is an endocrinologically active neoplasm. The neoplastic cells secrete excessive amounts of renin, which further activates the renin-angiotensin-aldosterone system (RAAS) in a secondary manner. The condition is classified as either typical or atypical. The most typical clinical symptoms are uncontrollable hypertension and hypokalemia, which lead to a series of concomitant manifestations, such as headache, weakness, blurred vision, nausea and vomiting. It is evident that these present their own unique clinical and pathological characteristics. In this paper, we present a case of atypical JGCT, with a clinical presentation dominated by poorly controlled recurrent hypertension. We also perform a review of the relevant published literature.

## Case report

**Figure 1:**
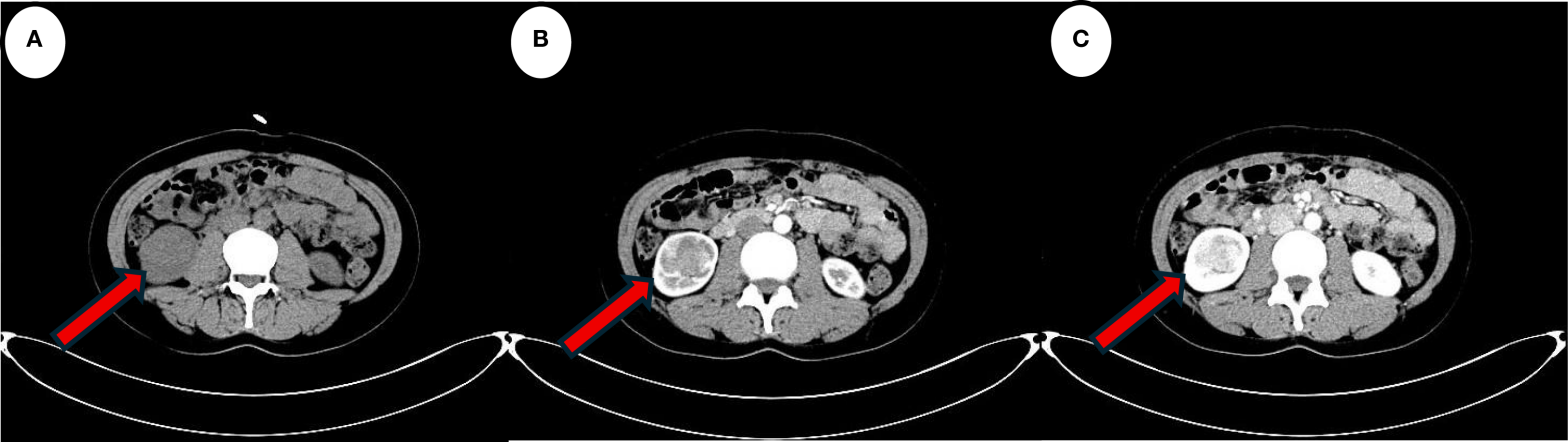
The red arrow indicates the lesion. **(A)** Computed tomography scan suggests a localised hypodense occupancy of the right kidney. **(B)** A small amount of enhancement is seen in the cortical phase of the enhanced scan. **(C)** Enhancement is more obvious in the medullary phase. The features are consistent with the typical presentation of JGCT imaging.

**Figure 2:**
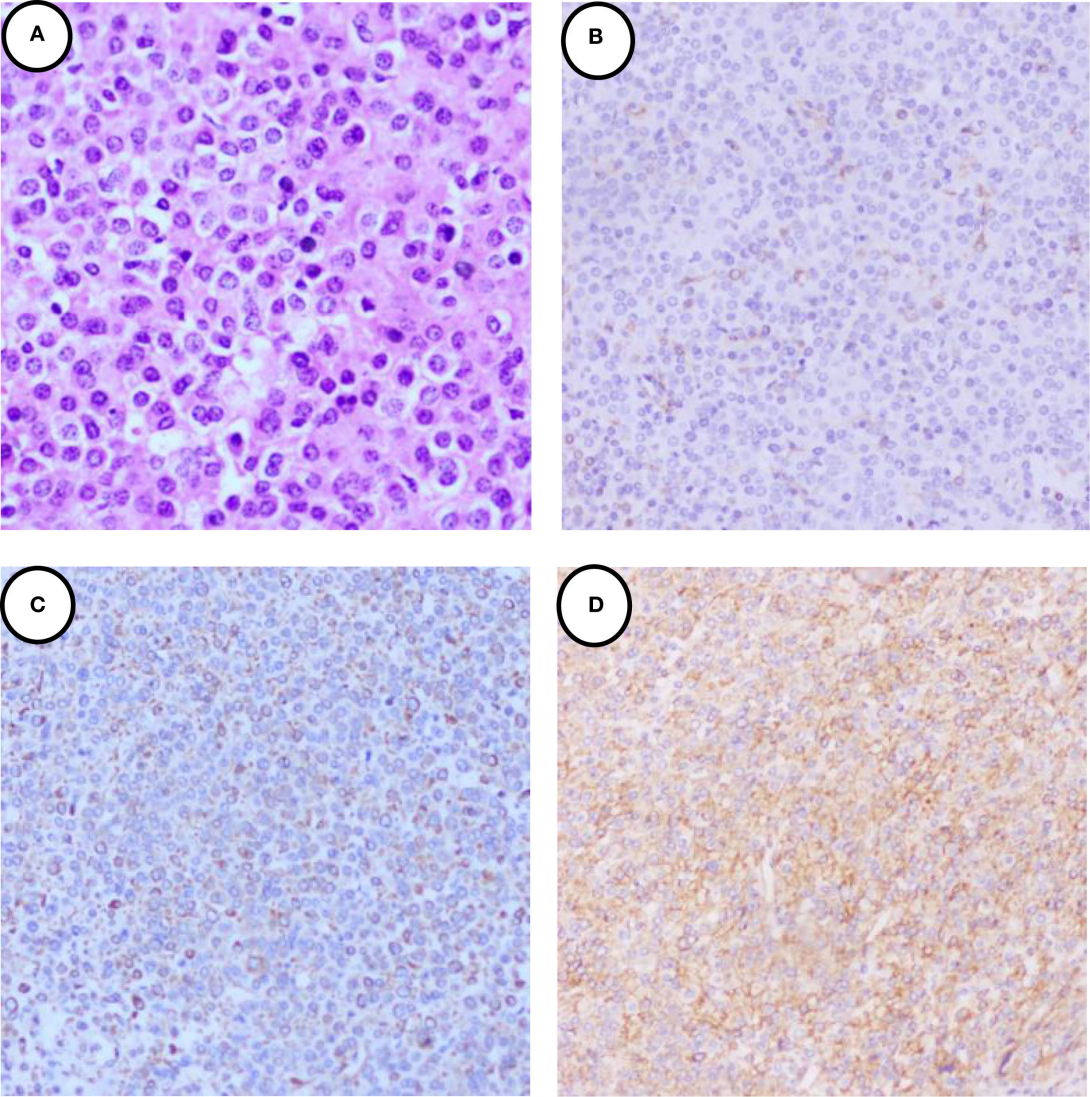
Immunohistochemical staining of the JGCT. **(A)** 40×H&E staining revealed that the tumor cells exhibited a round and polygonal morphology with homogeneous cytoplasm, and lacked clear cell borders and heterogeneity. **(B)** showed the tumor cells were cytoplasmic positive for CD34. **(C)** showed the tumor cells were cytoplasmic positive for Vimentin. **(D)** showed the tumor cells were cytoplasmic positive for SMA.

**Figure 3:**
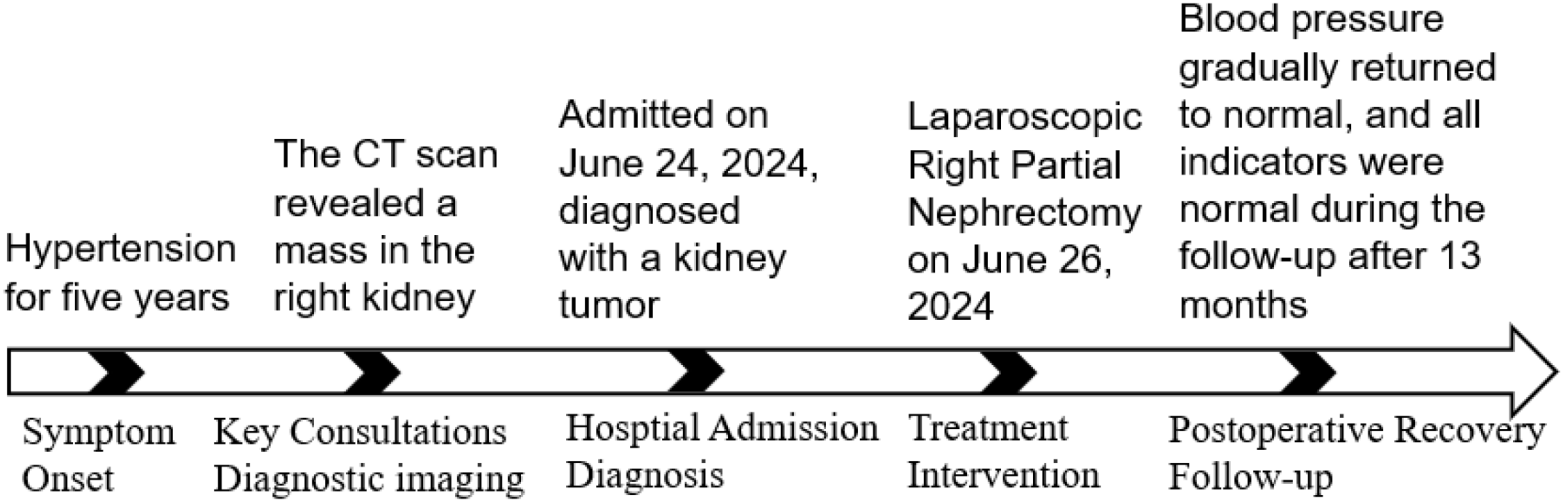
Visual timeline of the patient’s course.

A 39-year-old female patient was admitted to the Department of Cardiovascular Medicine at the Affiliated Hospital of Zunyi Medical University due to inadequate blood pressure control. To exclude the possibility of secondary hypertension, she underwent bilateral adrenal computed tomography scanning and enhancement. The results indicated that the body of the left adrenal gland exhibited slight thickening, while the right adrenal gland did not demonstrate any abnormalities in size, morphology, or density (**Figures 1A–C**). No abnormal reinforcement was identified in the enhancement scans. The right renal mass with obvious inhomogeneous enhancement measured approximately 61×35 mm. The patient was admitted to the urology department for further evaluation and treatment. The patient’s blood pressure had previously been inadequately controlled during pregnancy 10 years ago. The possibility of hypertension in pregnancy had been considered in the external hospital. The patient’s blood pressure remained unstable after the end of the pregnancy. The patient has been regularly monitored and treated with oral antihypertensive medication to date. The patient had a medical history significant only for hypertension and one previous pregnancy. She denied any family history of genetic disorders. Both of her parents were healthy and had no history of hypertension. After admission to the urology department, her blood pressure ranged from 155–165/83–88 mmHg. Physical examination revealed no significant abnormalities, particularly with respect to the cardiovascular, renal, and neurological systems. Following a thorough preoperative evaluation that ruled out any surgical contraindications, the patient underwent laparoscopic partial right nephrectomy. Postoperative pathology (see **Figures 2A–D**) showed the presence of tumor in the right kidney and the tumor cells appeared in a circular polygon shape with HE staining, which was confirmed by immunohistochemical staining. The following markers were observed: CD34 (+), Vimentin (+), ERG (scattered +), SMA (partially +), GATA3 (partially +), CD10 (partially +), CD117 (scattered +), S-100 (scattered +), Syn (scattered +), Ki-67 (2%+). The following markers were found to be negative: CK, CK5/6, CK7, HMB45, CA9, Ksp-cadherin, Melan-A, p63, PAX-8, RCC, TFE3, CD56, CgA, CK18, CK8, NSE, Desmin. Subsequent to the surgical procedure, the patient’s blood pressure exhibited a gradual return to its norm. Following a comprehensive analysis of the patient’s clinical manifestations, imaging findings, and pathological results, a diagnosis of JGCT was rendered. It is noteworthy that the patient’s blood pressure has been effectively managed since the postoperative follow-up. During the 13-month outpatient follow-up, the patient reported well-controlled blood pressure and had discontinued oral antihypertensive medications. Repeated laboratory tests showed that renal function and serum potassium levels remained within normal limits. Follow-up abdominal CT and abdominal ultrasound examinations showed no evidence of tumor recurrence. In order to enhance the clarity of the case and the narrative flow, we have added a visual timeline of the patient’s course of illness (**Figure 3**).

## Discussion

JGCT is an extremely rare renal tumor, accounting for a very low proportion of all renal tumors (3). The cellular origin of JGCT is a focus of debate in the academic community, and the current trend is that it is a special group of cells differentiated from arterial smooth muscle cells during embryonic development of the body, which possesses a unique endocrine function due to its ability to secrete nephrin, based on the dual phenotypic characteristics of myogenic contractile properties and endocrine activity at the same time (4). Myoendocrine cells have been defined based on their dual phenotypic characteristics of myogenic contractile properties and endocrine activity. This provides an important morphofunctional basis for the in-depth analysis of its pathophysiological mechanisms. The following is a summary of cases of benign biological behavior of JGCT reported in the literature over the past decade (**Table 1**). The previously published biological behaviors of JGCT exhibit malignancy, as summarized in **Table 2**. The majority of JGCT are benign tumors that secrete large amounts of renin, which leads to secondary activation of the RAAS system, causing long-term uncontrollable hypertension in patients. Radical surgery can be a curative treatment; however, the biological behavior of JGCT showing malignant manifestations has also been reported in the past. Notwithstanding the potential for radical surgery to engender a positive outcome, there have been antecedent reports of JGCT manifesting malignant biological behavior. For instance, Duan et al. (5) documented a case of paraglomerular cell tumor invading the renal vein and developing bilateral lung metastases five years later, with a maximum diameter of the mass of 15 cm, deep vein and vena cava invasion, and necrosis of the tumor. Beaudoin et al. (6) reported a case of a 51-year-old female JGCT patient presenting with tumor invasion of blood vessels and a mass size of 9.8cm x 8.5cm x 7.3cm; Shera et al. (7) reported a case of an 8-year-old boy with paraglomerular paraganglioma recurring in the renal fossa 1 year after left nephrectomy, with an initial mass size of 8cm x 8cm and a tumor recurrence mass size of 5cm x 4cm with vascular and peripheral invasion and active nuclear division; Cucchiari et al. (8) reported a case of a 50-year-old man with paraglomerular cell tumor with multisite involvement in the kidney, liver, and spleen, and who underwent surgery and systemic therapy, and the disease progressed during treatment; Hagiya et al. (9) reported a JGCT case with atypical pathological features, in which the tumor invaded the renal vein, lymphatic and vascular invasion, with no signs of recurrence or metastasis at 14 months of follow-up; Munakata et al. (10) diagnosed a 74-year-old male patient with a tumor that did not appear to be metastatic, but postoperative pathology suggested that the tumor had massive necrosis and mitotic figures, and the patient survived for 9 months; Geisler et al. (11) diagnosed a rare young male patient with malignant JGCT and suggested that GATA3 positivity could be helpful in the diagnosis of other diseases; Sakiyama et al. (12) reported a case of a 7 year old boy with JGCT of the right kidney and multiple pulmonary metastases at the time of presentation; the metastatic lesions were pathological as those of the kidney, and the patient had no recurrence at 2 years of follow up. In cases of malignant biological behavior associated with JGCT, surgical intervention alone may not yield the desired outcomes, necessitating close monitoring and the development of a personalized systemic treatment plan.

**Table 1:** Summary of benign JGCT cases over the past decade.

Case no.	First author (year)	Age (years) /sex /side	Clinical symptoms	Treatment	Pathology	Follow up (month)
1	Vidal-Petiot, et al. (2015) (40)	20/F/L	HBP, Hypokalemia	LLPN	JGCT	3
2	Torricelli, et al. (2015) (30)	25/F/R	HBP, Hypokalemia, Headache	LRPN	JGCT	2
3	Gil, et al (2015) (41)	19/F/L	HBP	PCT-RFA	JGCT	1
4	Sierra, et al. (2015) (42)	19/F/L	HBP, Hypokalemia	LLN	JGCT	NR
5	Yang, et al (2016) (43)	29/F/L	HBP, Headache	LLPN	JGCT	30
6	Soni, et al (2016) (44)	40/M/R	HBP	RPN	JGCT	NR
7	Wu, et al (2016) (45)	62/F/R	HBP, Hypokalemia, Hypertensive heart disease	LRRN	JGCT	18
8	Ma, et al (2016) (46)	32/M/R	No clinical manifestations	LRPN	JGCT	NR
9	Pacilli, et al. (2016) (47)	14/F/R	HBP	LRPN	JGCT	6
10	Miyano, et al. (2016) (48)	15/F/L	No clinical manifestations	LLPN	JGCT	12
11	Xue, et al (2017) (49)	31/F/L	HBP	LRAD LLPN	ACA JGCT	6
12	Daniele, et al. (2018) (50)	23/F/R	HBP, Hypokalemia	LRPN	JGCT	24
13	Nunes, et al. (2018) (51)	22/F/R	HBP, Proteiuria	LRPN	JGCT	4
14	Walasek, et al. (2019) (52)	25/F/R	HBP, Headache, Hypokalemia	ORPN	JGCT	12
15	Nassiri, et al. (2020) (53)	29/F/L	HBP, Headache, Hypokalemia	RALPN	JGCT	0.5
16	Krishnan, et al. (2020) (54)	41/M/L	HBP, HF, Hypokalemia, Headache, Difficulty in breathing	RALPN	JGCT	NR
17	Jiang, et al (2020) (55)	29/F/L	No clinical manifestations	LRN	JGCT	NR
18	Dong, et al. (2020) (56)	29/F/L	No clinical manifestations	RALPN	JGCT	6
19	Mezoued, et al. (2020) (57)	18/F/L	HBP, Hypokalemia	LLPN	JGCT	1
20	Phillis, et al. (2021) (58)	13/F/L	HBP, Palpitation, Difficulty in breathing	RALPN +LND	JGCT	9
21	Papez, et al. (2021) (59)	16/M/L	Painful hard lumps in the limbs, HBP, Hypokalemia Proteiuria	LRN	SCH and JGCT	12
22	Pan, et al (2021) (60)	39/F/L	HBP, Dizziness	LLPN	JGCT	3
23	Ueda, et al (2021) (61)	17/M/R	HBP, HF(DCM)	LRPN	JGCT	NR
24	Skarakis, et al. (2022) (62)	33/M/R	HBP, Hypokalemia, Headache	ORRN	JGCT	6
25	Quach, et al. (2022) (63)	19/F/L	HBP, Weakness in the upper limbs	LPN	JGCT	14
26	John, et al (2022) (64)	35/F/R	HBP, Hypokalemia	RARPN	JGCT	3
27	Mondschein, et al. (2022) (65)	21/F/L	HBP, Hypokalemia	LLPN	JGCT	12
28	Ruan, et al (2023) (66)	12/F/R	HBP, Chest distress	LRPN	JGCT	50
29	Aouini, et al. (2023) (67)	25/M/L	HBP, Headache, Hemorrhagic stroke	LRN	JGCT	12
30	Pompozzi, et al (2023) (68)	14/F/R	HBP, Hypokalemia, Metabolic alkalosis	LRPN	JGCT	NR
31	Fu, et al (2023) (69)	28/F/R	HBP, Nausea, Dizziness, Headache	LRPN	JGCT	18
32	Sekhon, et al. (2024) (70)	15/F/R	HBP, Hypokalemia, Hyponatremia, Headache	LRRN	JGCT	NR
33	Yan, et al (2024) (71)	47/F/R	HBP, Hypokalemia, Headache	LRPN	JGCT	24
34	Chen, et al (2024) (72)	27/F/L	HBP, Hypokalemia	LLPN	JGCT	2
35	Chaker, et al. (2025) (73)	35/M/R	HBP, Headache	ORPM	JGCT	24
36	Woods, et al. (2025) (74)	37/M/R	HBP, Hypokalemia	RPN	JGCT	NR

M, male; F, female; L, left; R, right; NR, No Reported; HBP, High Blood Pressure; HF, Heart Failure; DCM, Dilated Cardiomyopathy; LLPN, Laparoscopic Left Partial Nephrectomy; LRPN, Laparoscopic Right Partial Nephrectomy; LLRN, Laparoscopic Left Radical Nephrectomy; LRRN, Laparoscopic Right Radical Nephrectomy; RALPN, Robot-assisted Left Partial Nephrectomy; RARPN, Robot-assisted Right Partial Nephrectomy; OLPN, Open Left Partial Nephrectomy; ORPN, Open Right Partial Nephrectomy; OLRN, Open Left Radical Nephrectomy; ORRN, Open Right Radical Nephrectomy; LPN, Left Partial Nephrectomy; RPN, Right Partial Nephrectomy; LRN, Left Radical Nephrectomy; RRN, Right Radical Nephrectomy; PCT-RFA, Percutaneous Computed Tomography-Guided Radiofrequency Ablation; LRAD, Laparoscopic Right Adrenalectomy; LND, Lymph Node Dissection; SCH, Spindle Cell Hemangioma; ACA, Adrenocortical Adenoma.

**Table 2:** Summary of previous malignant cases.

Case no.	First author (year)	Age (years) /sex /side	Clinical symptoms	Treatment	Pathological evidence	Metastasis/recurrence	Follow up (month)	Reference
1	Duan et al (2004)	52/M/R	Hematuria	RRN Thoracotomies	Renal vein invasion	Bilateral Lung	72	(5)
2	Beaudoin et al (2008)	51/F/R	HBP, HK	RRN	Venous invasion	None	36	(6)
3	Shera et al (2011)	8/F/L	HBP, HK	LRN	Vascular invasion, Active nuclear division	Local recurrence	12	(7)
4	Cucchiari et al (2013)	50/F/L	HBP, HK, Decrease in weight	LPN, Splenectomy, Hepatic segmentectomy, Adjuvant chemotherapy	Necrosis, Vascular invasion	Spleen, Liver	44	(8)
5	Hagiya et al (2020)	31/M/L	HBP, HK	LRN	Renal vein, lymphatic and vascular invasion	None	14	(9)
6	Munakata et al (2018)	74/M/R	HBP, Proteiuria Hematuria	LRRN	Massive necrosis, Mitotic figures	None	9	(10)
7	Geisler et al (2022)	23/M/R	HBP, HK	RARPN	Mitotic activity	None	3	(11)
8	Sakiyama et al (2022)	7/M/R	HBP, Proteiuria	RRN, Chemotherapy, Thoracotomies	Tumor necrosis	Bilateral Lung	24	(12)

M, male; F, female; L, left; R, right; LRN, Left Radical Nephrectomy; RRN, Right Radical Nephrectomy; HBP, High Blood Pressure; RARPN, Robot-assisted Right Partial Nephrectomy; HK, Hypokalemia.; LRRN, Laparoscopic Right Radical Nephrectomy; LPN, Left Partial Nephrectomy.

The typical presentation of JGCT is characterized by hypertension, hypokalemia, renin hypersecretion and secondary aldosteronism. However, the accurate diagnosis of atypical patients is challenging and should be considered when there is a combination of a renal mass and hypertension or hypokalemia. JGCT is rare, and therefore the differential diagnosis of JGCT is critical in smaller hospitals with inexperienced clinicians, based on the combination of clinical presentation, imaging and pathology. In order to differentiate this disease, clinical manifestations, imaging data and histology are required. Some of the common diseases that need to be differentiated are renal cell carcinoma, primary aldosteronism, angiomyolipoma, renal cysts and nephroblastoma (**Table 3**). JGCT is prevalent in adolescents and young adults, with a higher proportion of females (13), and its clinical presentation is as described above. Patients with atypical JGCT may have only a single symptom, such as hypertension or hypokalemia. Previous studies have concluded that computed tomography (CT) and computed tomography contrast are useful for the JGCT, having high diagnostic sensitivity and specificity (14). CT shows a low-density occupancy, and enhanced CT often suggests that the tumor has no significant enhancement in the cortical stage, and the CT value of tumors in the parenchymal stage is higher than that of the cortical stage, so that the tumors in the parenchymal and delayed stages are shown more clearly than those in the cortical stage (slow in progress and slow in regression) (15); however, as newer studies have been found to be useful, Magnetic resonance imaging (MRI) has shown a better status in detecting JGCT (16). JGCT is shown to be a well-defined lesion on MRI, with isosignal or low-signal areas on T1-weighted images and predominantly high-signal on T2-weighted images, with homogeneous or haloed, nodular high-signal on DWI, and progressive enhancement on enhancement (17). It is important to note that the aforementioned examinations are merely suggestive, and a definitive diagnosis of the tumor necessitates a pathological examination. Light microscopic HE staining of JGCT revealed tumor cells with uniformly rounded, polygonal or spindle-shaped morphology, eosinophilic cytoplasm or light staining, unclear cell borders, small and regular nuclei, fine chromatin, and rare karyorrhexis. The histological structure exhibited solid sheets, nests or beams of tumor cells, and the interstitium was characterized by a high density of thin-walled blood vessels. Immunohistochemistry (IHC) phenotypes are specific and pivotal in confirming diagnoses. It is evident that the following positive markers (18) should be considered: The positivity of CD34 (+) is indicative of vascular endothelial markers (i.e. diffuse strong cytoplasmic positivity of the tumor cells, characteristic of the presentation), SMA (+) is suggestive of myxoid differentiation, and vimentin (+) is suggestive of mesenchymal origin. The most confirmatory marker is Renin. The most significant confirmatory marker is Renin, which demonstrates granular cytoplasmic positivity. Among the negative markers, CK (-), PAX8 (-), RCC (-), and HMB45 (-) have been found to be effective in excluding other renal tumors (19). Although Renin was not performed in this case, the patient was finally diagnosed as a case of JGCT by means of a differential diagnosis of the patient’s clinical presentation, imaging and a number of immunohistochemical positive, weakly positive and negative expression results.

**Table 3:** Similarities and differences among common diseases.

Diseases	RCC	PA	AML	RC	WT	JGCT
Clinical Features	Hematuresis Osphyalgia	HBP Hypokalemia	Osphyalgia	No clinical Features	Abdominal mass Children	HBP Hypokalemia
Imaging	Rapid wash-in and wash-out enhancement	Adrenal space occupying lesion	Uneven strengthening	Non-enhanced cystic lesion	Uneven strengthening	Gradual reinforcement
Histopathology	CAIX(+)/CK7(+)	CYP11B2(+)	HMB-45(+)	Simple epithelium	WT1(+)	Renin (+)
Laboratory examination	No Specificity	HA+LR	No Specificity	No Specificity	No Specificity	HA+HR
Treatment	SM+ TT/IT	SM+Medication	SM	SM	SM+CR	SM

RCC, Renal cell carcinoma; PA, primary hyperaldosteronism; AML, Angiomyolipoma; RC, Renal cyst; WT, Wilms’s tumor; JGCT, Juxtaglomerular Cell Tumor; HBP, High Blood Pressure; SM, Surgical Management; TT, Targeted Therapy; IT, Immunotherapy; CR, Chemoradiotherapy; HA, High Aldosterone; LR, Low Renin; HR, High Renin.

It is noteworthy that several subtypes of renal cell carcinoma are characterized by the absence of clinical manifestations in the early stage, with a predilection for middle-aged and elderly patients. The typical presentation of the condition includes symptoms such as hematuria, lumbar pain and abdominal mass. However, with the increased prevalence of medical checkups in recent times, the number of patients exhibiting this triad has decreased. When it does occur, it may be indicative of advanced tumor growth. Furthermore, a computed tomography scan may reveal a rounded mass in the renal parenchyma. The distinction lies in the temporal profile of the enhancement scan for renal clear cell carcinoma, which manifests as a ‘fast in and fast out’ phenomenon, characterized by a substantial blood supply. In the arterial phase, the enhancement is conspicuous, and the tumor’s density is lower than that of the normal renal parenchyma. In the venous phase, the enhancement diminishes further, and the contrast with the surrounding tissues reveals an even lower density. The enhancement of papillary renal carcinoma is weaker and shows slow progressive enhancement, while that of chromophobe cell tumor is in between (20).

Contrast-enhanced ultrasound (CEUS) is a real-time, dynamic imaging modality that can demonstrate the entire process of the contrast agent from its entry into the renal tumor and renal parenchyma until its subsidence. It also allows for observation of the tumor’s blood supply in real time. CEUS has been shown to have a role in differentiating cystic and solid masses in the kidney. Previous studies have reported that CEUS has higher sensitivity and specificity than enhanced CT in the differential diagnosis of hemorrhagic cystic renal lesions and solid tumors (21, 22). It has been reported in the literature that CEUS has a higher level of sensitivity and specificity than enhanced CT in the differential diagnosis of hemorrhagic renal cystic lesions and solid tumors (21, 22).CEUS is a more effective tool in the differential diagnosis of renal carcinoma, as it is typically fast-acting with high enhancement, and its performance differs significantly from that of renal tumors, which are chronic and with low enhancement (23). The rationale behind the examination of the low enhancement and chronicity of JGCT enhancement imaging is that the substantial renin output results in vasoconstriction, leading to the constriction of the lumen and, consequently, diminished blood flow (24).

As a renin-secreting tumor, elevated renin levels often lead to secondary aldosteronism, which in turn causes uncontrollable hypertension. This condition must be differentiated from primary aldosteronism (25, 26), where renin levels are often normal or reduced (27), and imaging suggests adrenal gland occupancy, which is responsible for about one-fifth of refractory hypertension (28). JGCT secretes excessive renin, and renin levels obtained by routine venous blood collection often lead to false negatives. It has been demonstrated that renin levels obtained from routine venous blood collection frequently result in false negatives. Consequently, previous studies have advocated that selective deep vein blood collection is of greater diagnostic value in identifying bilaterally different hormone levels (29). However, recent studies have indicated that renal vein blood collection is impractical in clinical settings, challenging to execute, and lacks sensitivity and specificity (27). Unfortunately, although the patient underwent bilateral adrenal CT scanning upon admission, which revealed mild left adrenal gland thickening, no space-occupying lesion was detected in the adrenal glands. Consequently, not all preoperative indicators for elevated blood pressure were thoroughly evaluated. Only routine preoperative preparations were completed to rule out surgical contraindications, and surgery was performed specifically to clarify the nature of the renal mass. Therefore, for patients with an established diagnosis of hypertension, if CT indicates adrenal gland thickening, adrenal hormone level testing should be performed to avoid poorly controlled blood pressure after JGCT resection and the need for secondary surgery.

The treatment option for JGCT is laparoscopic partial nephrectomy as the first choice (27, 30–32), but surgical treatment is absolutely feasible only after the diagnosis of secondary hypertension due to the source of the renal lesion has been made after characterization, localization, and etiology. Preoperative control of the patient’s blood pressure and active correction of hypokalemia are required (26), and PN can greatly preserve the function of the kidney itself and cure uncontrollable hypertension and electrolyte disorders (25, 27). A case of JGCT with long-term recurrent hypertension and renal insufficiency has been reported in the literature, and renal transplantation was performed due to renal failure after removal of the tumor (33), and there was also a patient with JGCT combined with membranous glomerulonephritis, whose blood pressure was normalized in a short period of time after the operation, but uncontrollable hypertension reappeared after one and a half years of follow up (34), so it is not that a single operation can solve the whole problem, and it is necessary to consider the patient’s condition comprehensively in order to develop an individualized plan for the patient. Therefore, not all problems can be solved by a single surgery, and comprehensive consideration of the patient’s condition is still needed, with a view to individualizing the treatment plan.

In summary, complete surgical excision remains the only curative approach and the gold standard for JGCT. The choice of surgical procedure depends on the size and location of the renal mass, as well as its relationship with blood vessels or the collecting system. Laparoscopic/robot-assisted partial nephrectomy is currently the preferred approach, as reflected in most previously published cases (**Table 1**), allowing complete tumor removal while preserving maximal functional renal parenchyma. Thorough preoperative preparation is essential, including strict control of blood pressure, management of complications, and correction of electrolyte imbalances. Postoperatively, blood pressure and potassium levels may not normalize immediately; antihypertensive and potassium supplementation therapies should be continued and tapered gradually based on the patient’s clinical response.

It has been established through previous studies that the occurrence of JGCT is associated with the expression of certain oncogenes located on chromosomes 4 and 10, and the loss of specific oncogenes on chromosomes 9, 11, and X (35–37).The primary treatment for JGCT remains surgical intervention; however, patients with metastases or those unable to undergo surgery may require systemic therapy. In 2023, Treger et al. (38) identified the NOTCH1 rearrangement in JGCT, which can be targeted by existing NOTCH1 inhibitors for therapeutic targeting. In 2024, Lobo et al. (39) emphasized a specified role for the MAPK-RAS pathway by IHC and whole exome sequencing (WES), but the specific pathogenesis of JGCT still needs to be explored by large-scale genomics. Overall, patients with JGCT have an excellent prognosis. Successful surgical removal of the lesion typically leads to complete resolution or significant improvement of hypertension and electrolyte disturbances, as demonstrated in the present case where the patient’s blood pressure remained within the normal range and no hypokalemia was observed during follow-up. The vast majority of JGCTs are benign; however, very few cases exhibit malignant biological behavior (**Table 2**). Therefore, in the presence of features suggestive malignancy, close postoperative surveillance is crucial.

## Patient perspective

For years, I struggled with refractory hypertension and fatigue, unaware these were caused by a rare kidney tumor. The discovery of a renal lesion on CT was concerning, and the diagnosis felt overwhelming. However, surgery—recommended after my doctors identified a renal cause—led to a remarkable turnaround. My blood pressure normalized without medication, and repeated follow-ups confirmed normal results. This experience lifted a long-standing burden of anxiety. I am deeply grateful to my medical team and urge others with similar symptoms to seek timely, thorough evaluation. I provide full consent for my medical history to be shared and have signed the required informed consent form.

## Conclusion

The classic triad of hypertension, hypokalemia, and a renal mass is highly suggestive of JGCT, which, although mostly benign, requires prompt and definitive surgical management. Histopathology remains the gold standard for diagnosis, as biochemical and imaging findings are only supportive. Complete resection, preferably through nephron-sparing surgery, is curative and typically resolves the metabolic abnormalities. Thus, in patients presenting with this triad, JGCT should be strongly suspected. Unnecessary medical management or delayed intervention must be avoided. Referral to specialized centers with experience in renal tumors is recommended to ensure accurate diagnosis and timely operation, both critical for favorable outcomes.

## Data Availability

The raw data supporting the conclusions of this article will be made available by the authors, without undue reservation.
